# Mesenchymal Stem Cell Secreted-Extracellular Vesicles are Involved in Chondrocyte Production and Reduce Adipogenesis during Stem Cell Differentiation

**DOI:** 10.1007/s13770-022-00490-0

**Published:** 2022-11-08

**Authors:** Yu-Chen Tsai, Tai-Shan Cheng, Hsiu-Jung Liao, Ming-Hsi Chuang, Hui-Ting Chen, Chun-Hung Chen, Kai-Ling Zhang, Chih-Hung Chang, Po-Cheng Lin, Chi-Ying F. Huang

**Affiliations:** 1 Department of Biotechnology and Laboratory Science in Medicine, National Yang Ming Chiao Tung University, Taipei, 11221 Taiwan; 2 Institute of Biopharmaceutical Sciences, National Yang Ming Chiao Tung University, Taipei, 11221 Taiwan; 3 Department of Medical Research, Far Eastern Memorial Hospital, New Taipei City, 220216 Taiwan; 4 College of Management, Chung Hua University, Hsinchu, 30012 Taiwan; 5 Gwo Xi Stem Cell Applied Technology Co., Ltd., Hsinchu, 30261 Taiwan; 6 Department of Pharmacy, National Yang Ming Chiao Tung University, Taipei, 11221 Taiwan; 7 Department of Fragrance and Cosmetic Science, Kaohsiung Medical University, Kaohsiung, 80708 Taiwan; 8 GTESTing Laboratory, Hsinchu, 30261 Taiwan; 9 College of Biological Science and Technology, National Yang Ming Chiao Tung University, Hsinchu, 30010 Taiwan; 10 Department of Orthopedics, Far Eastern Memorial Hospital, New Taipei City, 22060 Taiwan; 11 Graduate School of Biotechnology and Bioengineering, Yuan Ze University, Taoyuan, 32003 Taiwan; 12 Department of Biochemistry, Kaohsiung Medical University, Kaohsiung, 80708 Taiwan; 13 School of Pharmacy, Kaohsiung Medical University, Kaohsiung, 80708 Taiwan

**Keywords:** Cartilage regeneration, Ultrafiltration, Extracellular vesicles, Mesenchymal stem cells

## Abstract

**Background:**

Extracellular vesicles (EVs) are derived from internal cellular compartments, and have potential as a diagnostic and therapeutic tool in degenerative disease associated with aging. Mesenchymal stem cells (MSCs) have become a promising tool for functional EVs production. This study investigated the efficacy of EVs and its effect on differentiation capacity.

**Methods:**

The characteristics of MSCs were evaluated by flow cytometry and stem cell differentiation analysis, and a production mode of functional EVs was scaled from MSCs. The concentration and size of EVs were quantitated by Nanoparticle Tracking Analysis (NTA). Western blot analysis was used to assess the protein expression of exosome-specific markers. The effects of MSC-derived EVs were assessed by chondrogenic and adipogenic differentiation analyses and histological observation.

**Results:**

The range of the particle size of adipose-derived stem cells (ADSCs)- and Wharton’s jelly -MSCs-derived EVs were from 130 to 150 nm as measured by NTA, which showed positive expression of exosomal markers. The chondrogenic induction ability was weakened in the absence of EVs *in vitro*. Interestingly, after EV administration, type II collagen, a major component in the cartilage extracellular matrix, was upregulated compared to the EV-free condition. Moreover, EVs decreased the lipid accumulation rate during adipogenic induction.

**Conclusion:**

The results indicated that the production model could facilitate production of effective EVs and further demonstrated the role of MSC-derived EVs in cell differentiation. MSC-derived EVs could be successfully used in cell-free therapy to guide chondrogenic differentiation of ADSC for future clinical applications in cartilage regeneration.

**Supplementary Information:**

The online version contains supplementary material available at 10.1007/s13770-022-00490-0.

## Introduction

Osteoarthritis (OA) is the most common chronic joint condition, affecting millions of people worldwide [1]. Because OA is a long-lasting joint injury, it most often occurs in the elderly [2]. Recently, the age at onset has shown a decreasing trend, with OA occurring in younger patients [3, 4]. In the majority of cases, the main causes of OA in young adults are excessive obesity and excessive exercise [5–7].

In recent clinical treatments, intra-articular (IA) injections of hyaluronic acid (HA) [8] and biopharmaceuticals such as platelet-rich plasma (PRP) or mesenchymal stem cells (MSCs) are used to reduce inflammation, repair articular cartilage defects, and restore joint mobility [9–12]. Studies have shown that IA injection is effective for treating arthritis because the paracrine substances of PRP and MSC play an important role [13]. The high concentrations of growth factors (GFs) from PRP secretion have been reported to modulate the progression of the inflammatory process and promote healing [14]. In addition to GFs and cytokines, it has recently been found that extracellular vesicles (EVs) derived from stem cells are important mediators with anti-inflammatory and chondroprotective effects [15]. These vesicles, including exosomes and microvesicles, can convey a multiplicity of signals by transferring functional RNAs and proteins [16–19]. It has been reported that EVs secreted from PRP could significantly inhibit the apoptosis of OA chondrocytes by activating the Wnt/β-catenin signaling pathway [20]. Moreover, OA chondrocytes treated with EVs from adipose-derived stem cells (ADSCs) can reduce matrix metalloproteinase activity and enhance the expression of type II collagen (COLII) and the anti-inflammatory cytokine interleukin-10 in OA chondrocyte treatment [21]. In preclinical models of OA, EVs isolated from bone marrow MSCs (BM-MSCs), synovial membrane, or induced pluripotent stem cells (iPSCs) were also shown to mediate cartilage tissue repair by increasing proliferation and were found to protect chondrocytes by inhibiting apoptosis and macrophage activation [22–25]. These findings suggest that EV-based therapy may have potential as a novel cell-free approach in regenerative medicine.

For stem cell treatment of cartilage damage, because the number of input stem cells is limited, the goal of our study was to generate MSC-EVs and to demonstrate the effect of additional EVs addition in ADSCs on the tissue differentiation process. In this study, we used adipose and umbilical cord Wharton jelly-derived stem cells as EV production tools, and replacement of cell culture medium with serum-free medium before EV collection is currently a common method of EV production. Next, EVs were isolated and concentrated using an ultrafiltration (UF) membrane with a molecular weight cut-off of 100 kDa. The results showed that adding EVs in ADSCs effectively stimulated COLII production and the chondrogenic differentiation capacity, but also reduced oil droplet production in the adipogenesis process. Thus, based on the potential role of EVs in promoting cartilage tissue generation, it may have clinical applications in regenerative medicine.

## Materials and methods

### Expansion of human MSCs

In this experiment, we used human MSCs from three healthy donors. Human ADSCs from two donors were tested for chondrogenic and adipogenic differentiation and one of ADSCs was used as a tool for EV production; WJ-MSCs (RM60596) were purchased from the Bioresource Collection and Research Centre (BCRC), Hsinchu, Taiwan, and were also used as an EV production tool. All cells were provided by Gwo Xi Stem Cell Applied Technology Co., Ltd. (Taipei City, Taiwan) and donors signed a consent form for cell donation.

The cells were cultured and maintained in keratinocyte serum-free medium (KSFM; Invitrogen-Gibco, Grand Island, NY, USA) supplemented with 10% (v/v) fetal bovine serum (FBS; HyClone, Logan, UT, USA), antioxidants *N*-acetyl-L-cysteine (Sigma-Aldrich, St. Louis, MO, USA), and L-ascorbic acid *2-*phosphate (Sigma-Aldrich). The cells were incubated at 37 °C with 5% CO_2_ until the monolayer of adherent cells reached 70–80% confluence. During the experiment, the serum-free medium (SFM) was keratinocyte serum-free medium containing *N*-acetyl-L-cysteine and L-ascorbic acid 2-phosphate, which was used as a production medium for collecting EVs. Exosome-depleted FBS (EV-depleted FBS) was purchased from Gibco (Invitrogen-Gibco). The cells were used for the study in the passages 5.

### Flow cytometry analysis

The ADSCs were seeded into a 175-cm^2^ flask at a density of 5 × 10^5^ cells per flask. After growth for four days, culture medium was replaced with serum-free media for 24 h, and then cells were harvested*.* The surface phenotypes of ADSCs were characterized using a BD Accuri C6 flow cytometer (Becton Dickinson, Franklin Lakes, NJ, USA), and cells were stained with antibodies against the human clusters of differentiation (CD): CD14, CD19, CD34, CD45, CD73, CD90, CD105, and HLA-DR (Becton Dickinson). Labeling was performed according to the manufacturer’s instructions.

### ADSC differentiation capability

ADSCs were seeded in 24-well plates at a density of 5000 cells/well in culture medium containing 10% FBS. After 24 h, the culture medium was removed, and cells were washed twice with Dulbecco's phosphate buffered saline (DPBS; Corning, Manassas, VA, USA) without calcium and magnesium. Cells were placed in SFM for 24 h. ADSCs were then induced to differentiate into adipogenic, osteogenic, and chondrogenic lineages using differentiation medium for 21 days.

#### Adipogenic differentiation

For adipogenic differentiation, ADSCs were incubated with PRIME-XV Adipogenic Differentiation SFM (Irvine Scientific, Santa Ana, CA, USA) containing 10% FBS, and the medium was exchanged every 3 days. On days 7, 14, and 21, the adipogenic differentiation of ADSCs was fixed in 4% formaldehyde solution (Sigma-Aldrich) for Oil Red O staining (ScyTek, BH Hague, Netherlands). Cells were observed under a light microscope [26].

#### Osteogenic differentiation

For osteogenic differentiation, PRIME-XV Osteogenic Differentiation SFM (Irvine Scientific) supplemented with 10% FBS was added to the wells for 21 days. On days 7, 14, and 21, the osteogenic differentiation of ADSCs was detected using an alkaline phosphatase (ALP) detection kit (Sigma-Aldrich) according to the manufacturer’s instructions. Stained cells were captured as images under a light microscope, as described previously [27].

#### Chondrogenic differentiation

ADSCs were cultured in PRIME-XV Chondrogenic Differentiation SFM (Irvine Scientific) containing 10% FBS for 21 days. After chondrogenic induction, the spheroid formation of ADSCs exhibited cartilage-like features. Chondrogenic pellets were subjected to histological staining. The pellets were fixed in 4% formaldehyde, dehydrated using alcohol, cleared with xylene (EMD; J.T. Baker, CAS 1330-20-7), and embedded in paraffin. Finally, paraffin-embedded tissue was cut into 4-μm-thick sections, placed on glass slides, and dewaxed. Following the methods described previously [28], Alcian blue staining (ScyTek) was used to determine the production of sulfated glycosaminoglycans (GAGs). Cells were observed under a light microscope and images were captured.

### Co-culture system

ADSCs were seeded in 12-well plates at a density of 5000 cells/well and the upper chamber of 8.0-µm Transwell plates (Corning) at a high density of 8 × 10^4^ cells. Cells were cultured overnight in a medium supplemented with 10% FBS and then rinsed twice with DPBS after removing the culture medium. The replacement culture medium of the upper chambers was culture medium containing 10% EV-depleted FBS, and the lower chambers were replaced with chondrogenic differentiation medium containing 10% EV-depleted FBS. The medium was refreshed every 3 days. On day 21, the efficiency of cartilage differentiation was assessed by histochemical staining (Alcian blue). Cells were analyzed under a light microscope (Olympus BX43, Tokyo, Japan). Commercially available EV-depleted FBS was purchased from Gibco (Life Technologies, Waltham, MA, USA).

### Isolation of EVs by UF

MSCs were collected from SFM, and two centrifugation processes were used to remove cell debris. First, the medium was centrifuged at 300×*g* for 5 min at 4 °C. After centrifugation, the supernatant was transferred to a new tube and then centrifuged at 4000×*g* for 20 min at 4 °C. After performing the differential centrifugation steps, EVs were collected from the supernatant. The supernatant was then filtered through a 0.22 μm filter (Merck Millipore, Billerica, MA, USA) to remove large vesicles. Amicon Ultra-15 with a molecular weight exceeding 100 kDa (Millipore) was used to remove the free protein, followed by centrifugation at 4000×*g*. The final volume depended on the subsequent steps. For standard preparation, medium was further concentrated 25-fold. The particle numbers and size were measured using NTA. The EV sample was kept at −80 °C until use.

### Nanoparticle tracking analysis

In this study, we used Nanoparticle Tracking Analysis (NTA, NanoSight NS300; Malvern Panalytical, Malvern, UK) for analysis of EV concentration and size distribution according to the manufacturer's instructions [29–31]. The collected medium was centrifuged to remove cell debris. Next, we used production medium diluted with water at a concentration range of 1 × 10^6^–1 × 10^9^ particles per milliliter for the NTA assay. One mL of dilution sample was injected into the sample chamber with sterile syringes, and particle concentration and size of samples were analyzed under a constant flow rate of 70 µL per minute at room temperature.

### Western blot analysis

We used Western blot analysis to detect the expression of positive markers of exosomes, i.e., transmembrane proteins Alix, TSG101, heat shock protein 70 (HSP70), CD9, and CD81. Calnexin in the endoplasmic reticulum, a negative marker, was used as one of the indicators of EVs purity. Culture supernatants were concentrated and transferred to sterile tubes, and then samples were incubated with ExoQuick-TC (System Biosciences, Palo Alto, CA, USA) at 4 °C for exosome precipitation. The exosome pellets were dissolved in the protein lysis buffer. After lysis, the samples were centrifuged to obtain the exosome proteins. Twenty-five μg of each sample was loaded onto a 10% sodium dodecyl sulfate polyacrylamide gel (SDS-PAGE). After electrophoresis, proteins were transferred to a PVDF membrane, probed with primary antibody followed by incubation with horseradish peroxidase (HRP)-coupled secondary antibody against the primary antibody. After incubating with the HRP-coupled secondary antibodies, visualization of the protein bands was performed by incubating with chemiluminescent substrate (Thermo Fisher Scientific) and the results were documented. Alix and Calnexin were purchased from Cell Signaling Technology (Danvers, MA, USA); the antibody against Tumor susceptibility gene 101 (TSG101) was purchased from Santa Cruz Biotechnology (Dallas, Texas, USA); HSP70 was purchased from BD Biosciences (Franklin Lakes, NJ, USA); CD9 and CD81 were purchased from R&D Systems, Minneapolis, MN, USA.

### Detection of MSC-derived functional EV via chondrogenic differentiation

During the chondrogenic differentiation process, ADSCs were cultured in EV-free FBS medium. EVs derived from ADSCs and WJ-MSCs were added to chondrogenic differentiation medium. The EV concentrations were 5 × 10^7^ and 5 × 10^8^, respectively. The medium was completely replaced every 3 days. At 14 days, cells were harvested, and the protein expression of COLII and cyclins (A2, B1, and D) (Cell Signaling Technology) was detected by chondrocyte induction. In addition, the protein expression levels were quantitated by ImageJ software. At 21 days, cartilage tissue was stained by Alcian blue, and the nuclei were stained with Nuclear Fast Red. Cells were observed under a light microscope and images were captured.

### Detection of MSC-derived functional EV via lipid droplets formation

During the differentiation process, ADSCs were cultured in adipogenic differentiation medium containing 5 × 10^7^ and 5 × 10^8^ EVs. The medium was replaced every 3 days. EVs were released from ADSCs and WJ-MSCs. On days 7 and 14, adipose-differentiated cells were stained by Oil Red O, and the accumulation of oil droplets was observed under a microscope. After imaging, oil red O-stained intercellular oil droplets were eluted with isopropanol and quantified by absorbance reading at 510 nm [26].

### RNA isolation and cDNA synthesis

Total RNA was extracted from cartilage tissue in EV-induced chondrogenic differentiation experiments using a GENEzol™ TriRNA Pure Kit (Geneaid) according to the manufacturer's instructions. RNA measurement was conducted by measuring absorbance at 260 nm and 280 nm using an Epoch™ Microplate Spectrophotometer (BioTek). One μg of total RNA in a volume of 20 μl was used to synthesize single-stranded cDNA using a High Capacity cDNA Reverse Transcription Kit (Applied Biosystems, Carlsbad, CA, USA). The reaction conditions were incubation at 25 °C for 10 min, followed by 37 °C for 120 min and the reaction was terminated by heating at 85 °C for 5 min. Finally, the cDNA products were stored at − 20 °C until use.

### Quantitative real-time PCR

To examine the expression patterns of chondrogenic-related genes in the ADSCs, quantitative real-time PCR (qRT-PCR) was performed. The gene-specific primers were provided by the Department of Medical Research, Far Eastern Memorial Hospital, Taiwan. The primer sequences used for qRT-PCR are listed in Table 1, and GAPDH was used as an internal control for normalization of gene expression. The qRT-PCR was conducted with iTaq™ Universal SYBR® Green Supermix (Bio-Rad) on a real-time PCR System. The PCR temperature cycling conditions were as follows: initial denaturation for 10 min at 95 °C, followed by 45 standard cycles: denaturation at 95 °C for 10 s, primer annealing for 10 s at 55 °C, and primer extension at 72 °C for 10 s. All reactions were conducted in triplicate.

**Table 1:** Primers used for quantitative real time polymerase chain reaction (qPCR) analysis

Gene	Primer	Sequence (5'–3')	Accession No.
Human GAPDH	Forward Reverse	GTCAAGGCTGAGAACGGGAA GCAGAGGGGGCAGAGATGAT	NM_002046.7
Human Aggrecan	Forward Reverse	CAGGAGAAGAAGAGGGTGGC CACTGGGGTATAGGCTGGTT	NM_001135.4
Human Collagen Type I	Forward Reverse	GCCTCCCAGAACATCACCTA TCAATCACTGTCTTGCCCCA	NM_000088.4
Human Collagen Type II	Forward Reverse	GATGCCACACTCAAGTCCCT GTCTCGCCAGTCTCCATGTT	NM_033150.3
Human SOX9	Forward Reverse	ACTACAGCGAGCAGCAGCAG AGCGGGGTTCATGTAGGTGA	NM_000346.4

### Statistical analysis

All data are shown as the mean and standard deviation. Levels of significance were analyzed using two-tailed paired Student’s *t*-test. Differences were considered statistically significant if *p* values were less than 0.05. *P* values less than 0.05 are denoted by “*”, *p* values less than 0.01 are denoted by “**”, and *p* values higher than 0.05 are denoted by “ns” (non-significant).

## Results

### Absence of EVs affects the ability to differentiate into various cell lineages

**Fig. 1:**
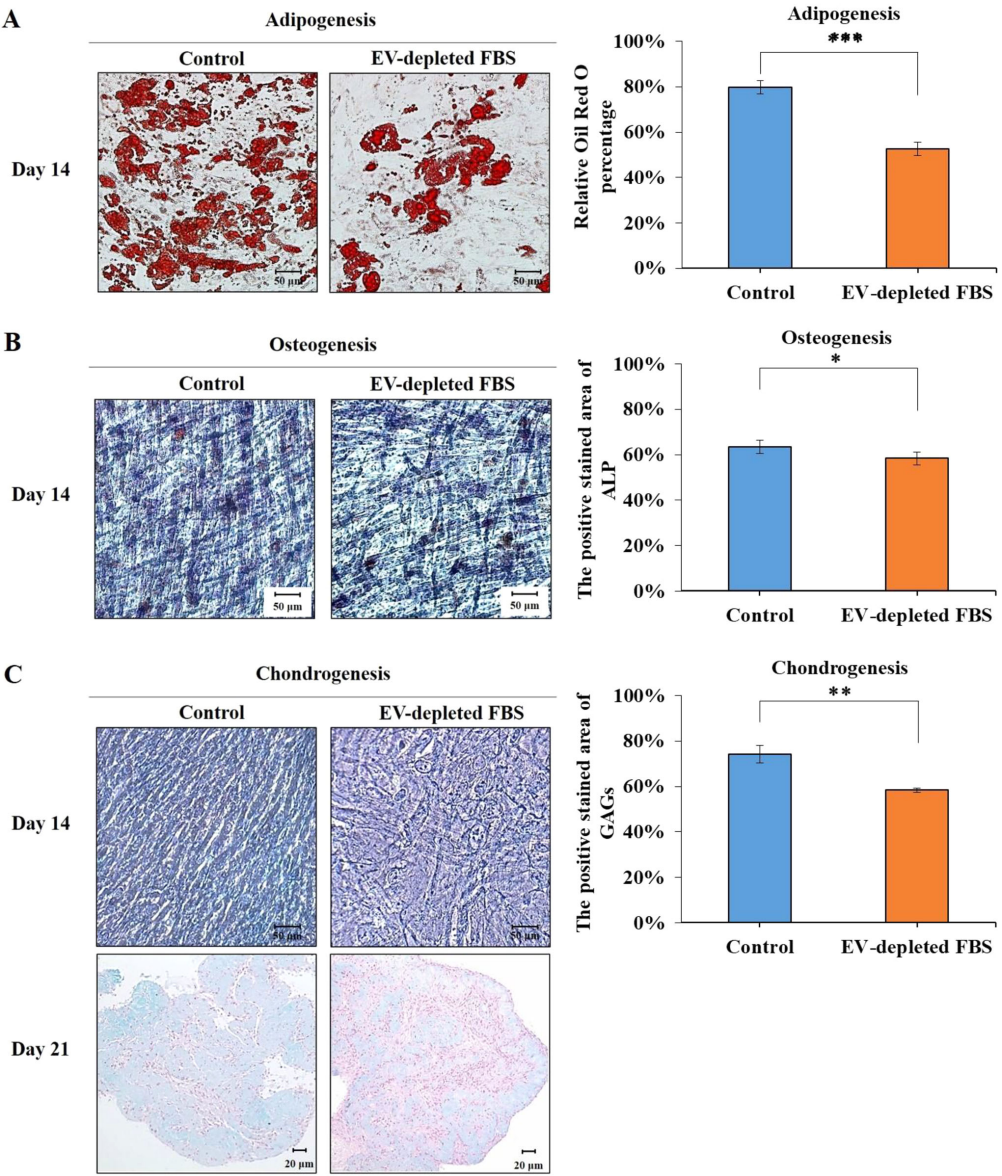
Effects of adipose-derived stem cells (ADSC) differentiation on specific lineages in differentiation medium with EV-depleted FBS. ADSCs from passage 5 were cultured in various differentiation induction media with 10% FBS. **A** ADSCs were cultured in adipogenic differentiation medium with EV-depleted FBS for 14 days and observed by microscopy, followed by Oil Red O staining. **B** Cells were cultured in osteogenic differentiation medium with EV-depleted FBS for 14 days and observed by microscopy, followed by alkaline phosphatase (ALP) staining. **C** For chondrogenic induction, cells were maintained in differentiation medium and observed by microscopy, followed by Alcian blue staining on day 14. The histology of paraffin-embedded tissue sections was evaluated on day 21 of chondrogenic differentiation. The differentiation quantifications of tissues were performed after 14 days of adipogenic, osteogenic differentiation, and after 21 days of chondrogenic differentiation, respectively. The percentage positive area was calculated using ImageJ software. The mean ± standard deviation (SD) levels of tissue differentiation in the three groups. *p < 0.05; **p < 0.01; ***p < 0.001, when EV-depleted FBS group was compared to the control group

To confirm the importance of EVs in cell differentiation, ADSCs were exposed to either normal FBS medium (control group) or EV-depleted FBS medium (experimental group). Cells were allowed to differentiate for 14 or 21 days and were then fixed and stained using the differentiation stain kit. The induction of adipogenic, osteogenic, and chondrogenic differentiation potential of ADSCs was observed under a light microscope. The results showed that oil droplet production diminished due to treatment with EV-depleted FBS (Fig. 1A). With respect to osteogenic differentiation, the density of stained ALP in the EV-depleted group was lower than in the control group (Fig. 1B). The cartilage extracellular matrix (ECM) is composed predominantly of a collagenous network and GAGs, such as chondroitin sulfate and HA [32, 33]. Alcian blue is one of a group of polyvalent basic dyes that bind and precipitate the sulfate and carboxylate groups from an aqueous solution [34]. Thus, Alcian blue can be used as a specific cartilage stain [28, 35]. Cartilage tissue sections were stained using Alcian blue staining, and GAG formation was reduced after EV-depleted FBS treatment (Fig. 1C). These findings indicate that the absence of EVs in the medium could affect cell differentiation, and that EVs may facilitate MSC differentiation.

### Secreted substances of ADSCs enhance chondrocyte differentiation capacity

**Fig. 2:**
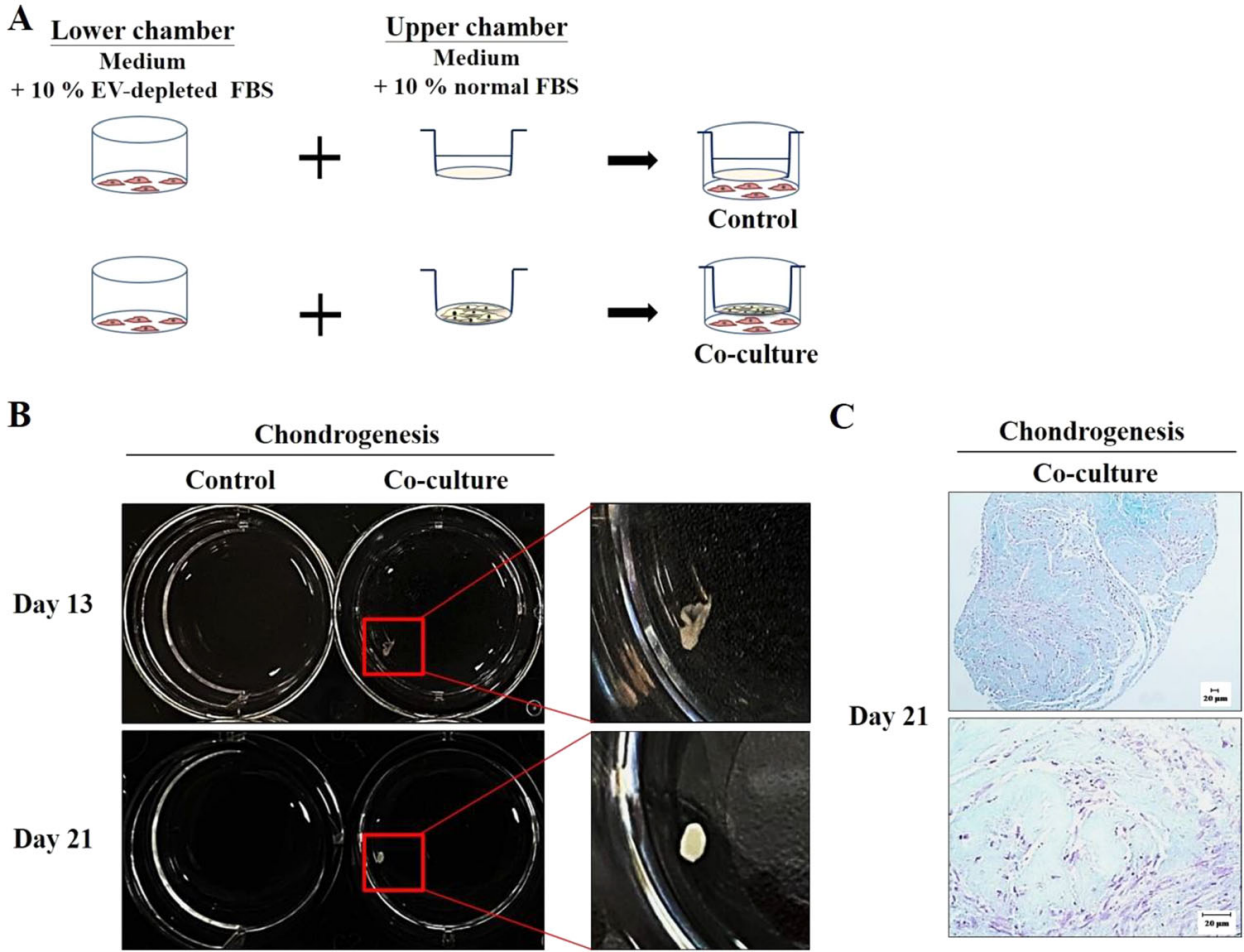
Effects of chondrogenic differentiation capacity by secreted substances of ADSCs. **A** Scheme of the experimental procedure. ADSCs were seeded at low density (5 × 10^3^ cells/well) in the lower well, and cells at high cell densities (8 × 10^4^ cells/well) were seeded in the upper chamber of 8.0-µm Transwell plates. The control group was the upper chamber without seeding cells. **B** The images recorded were used in the coculture system. Cartilage tissue was collected from the co-culture group. **C** The sliced cartilage tissue was stained with Alcian blue, and then cartilage differentiation status was obtained by microscopy. The nucleus was stained red–purple, and the cytoplasm was stained light pink

To determine whether secretory factors from ADSCs could promote ADSC differentiation into chondrocytes, we first compared the adipogenesis capacity of mesenchymal stem cells, ADSC and WJ-MSC, using the peroxisome proliferator-activated receptor gamma (PPARγ) expression level. The results showed that the adipogenic capacity of ADSCs was superior to that of WJ-MSCs through the protein expression of PPARγ (Supplementary Fig. S1). This result was also consistent with the results of other studies [36, 37] showing that ADSCs have strong adipogenic differentiation ability compared with other MSCs. Thus, we used ADSCs for further experiments. ADSCs were cultured in 10% EV-depleted FBS medium in the upper chamber using the co-culture system (Fig. 2A). The control group was not seeded with ADSCs in the upper chamber, but medium containing 10% EV-depleted FBS was also added. Then, chondrogenic differentiation-promoting factors in the upper chamber were secreted into the lower chamber. The results showed the secretion of soluble substances from ADSCs could induce the transformation of cells into chondrocyte spheroids at 13 days (Fig. 2B). GAGs are the major components of cartilage ECM and provide biological signals to stem cells and chondrocytes for cartilage development and functional regeneration [38, 39]. The results showed that sulfated GAG production was also detected in the ECM of cartilage tissue by histochemical assay (Fig. 2C). The cell differentiation capacity in the coculture group was better than in the control group, although the control group did not form chondrospheroids at 21 days (Fig. 2B). The results suggest that the paracrine effects of ADSCs may play a key role in the differentiation capacity of stem cells.

### EV production from MSCs does not alter their differentiation capability

**Fig. 3:**
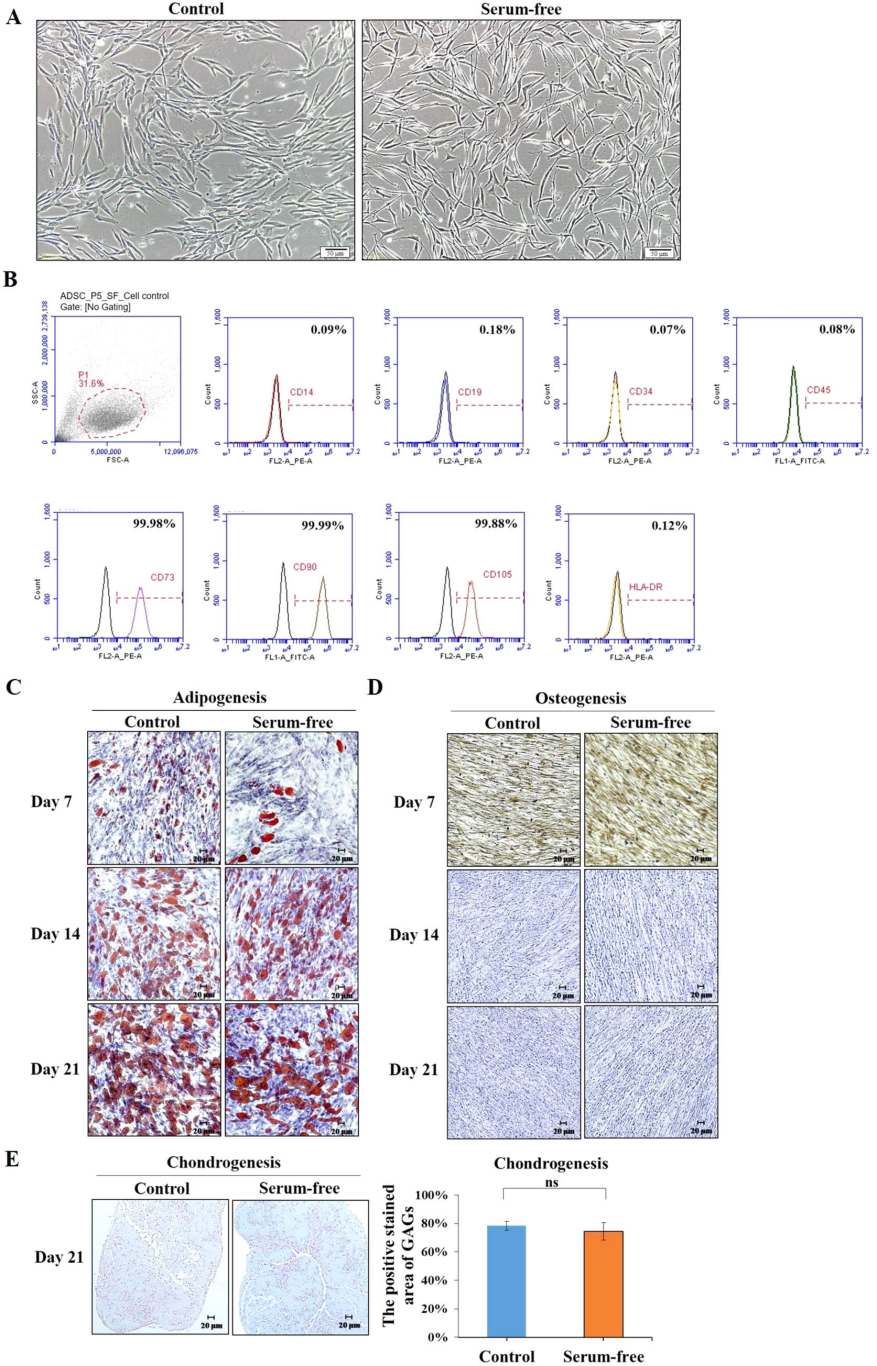
Monitoring of mesenchymal stem cell (MSC) characteristics under serum-free culture conditions. The control group was cultured in medium with 10% FBS, and the serum-free group was serum starved of ADSCs for 24 h before differentiation. **A** ADSC morphology was observed by microscopy. Scale bar, 50 µm. **B** Flow cytometry was used to determine surface marker expression, including CD14, CD19, CD34, CD45, HLA-DR, CD73b, CD90, and CD105. ADSC differentiation was induced in different types of differentiation media at passage 5. **C** Cells that had undergone adipogenic differentiation were fixed in 4% formaldehyde, and Oil Red O staining of ADSCs for adipogenic induction was observed by microscopy at 7, 14, and 21 days. **D** Cells were subsequently cultured in osteogenic differentiation medium for 7, 14, and 21 days and observed by microscopy, followed by ALP staining. **E** For chondrogenic induction, cells were maintained in differentiation medium for 21 days. Tissue sections were stained with Alcian blue and evaluated by microscopy. The mean ± standard deviation (SD) levels of chondrogenic differentiation in the three groups. A *p* value higher than 0.05 is denoted by “ns” (non-significant)

Serum-free conditions were used to induce EV production in MSCs. To assess whether the differentiation capability of ADSCs was affected by the EV collection process, cells were cultured in medium supplemented with 10% FBS for 4 days which was then replaced with SFM for 24 h. After 24 h, ADSC morphology was observed by light microscopy. Spindle-shaped ADSCs exhibited morphological characteristics that were thinner and longer compared to the control group (Fig. 3A). ADSCs were further characterized by flow cytometry to examine surface markers. As shown in Fig. 3B, there was positive expression of canonical markers (CD73, CD90, and CD105) but there were no negative markers (CD14, CD19, CD34, CD45, and HLA-DR). When ADSCs were pretreated for 24 h in SFM, the differentiation capacity of ADSCs was assessed by adipogenic, osteogenic, and chondrogenic assays. The results confirmed that the ability of ADSCs to differentiate into lipid production still existed during the long-term maintenance of adipogenic differentiation medium (Fig. 3C). The osteogenic and cartilage differentiation capacities did not decrease compared to the normal FBS group (Fig. 3D, E). Thus, this EV production method under serum-free conditions does not alter the characteristics of stem cells as examined by flow cytometry and stem cell differentiation assay. These results indicate that during the EV collection process, culturing under SFM condition for 24 h does not alter MSC physiological function, which suggests that under this condition, MSC-derived EV function and quality are likely not affected.

### EV quantification and characterization

**Fig. 4:**
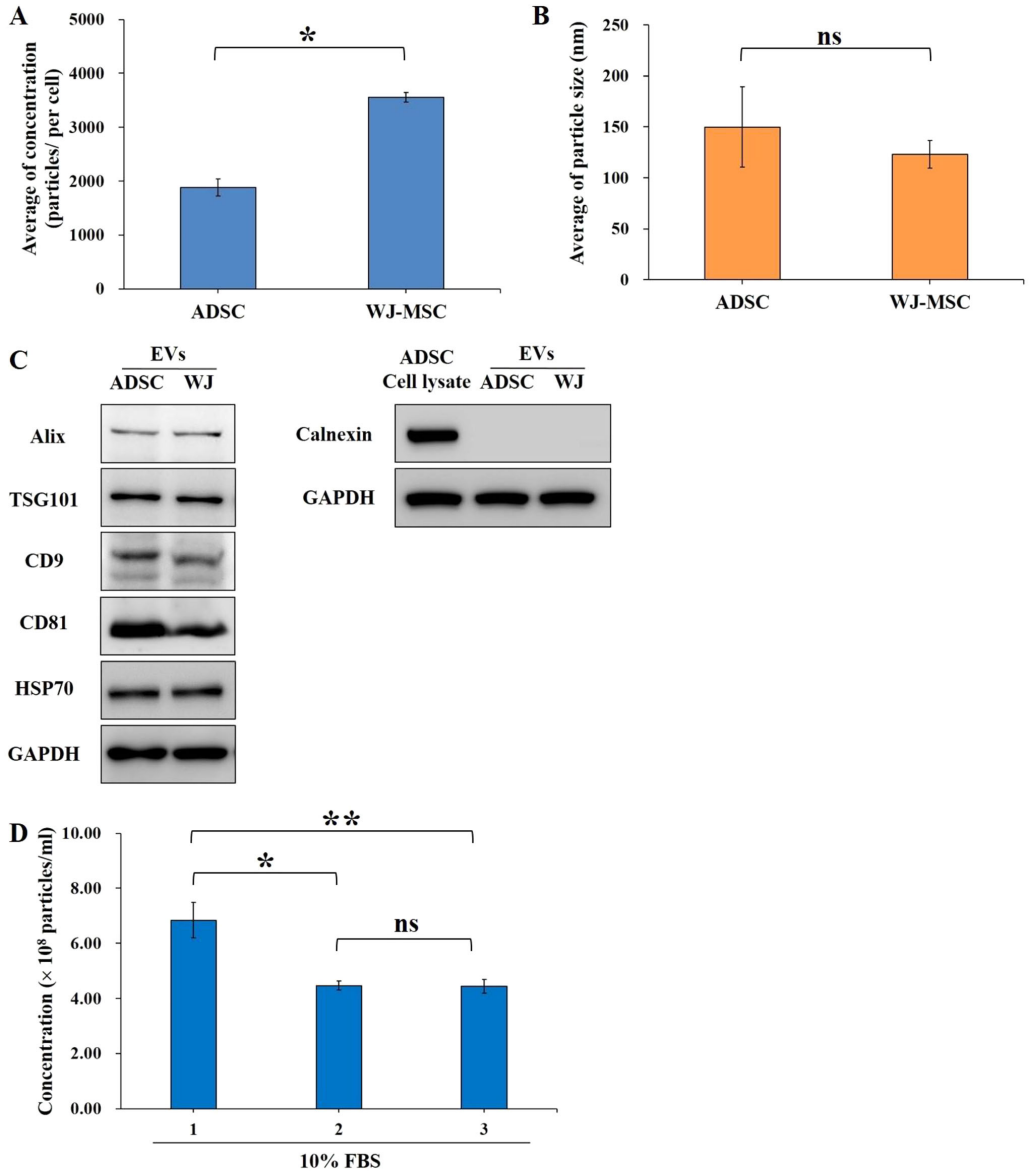
Extraction and identification of MSC-derived EVs. **A** Using NTA, the EV production yield from ADSCs and WJ-MSCs was analyzed by the average particle numbers in each cell. **B** The average size of EVs was determined by NTA. **C** The protein levels of EV markers, including Alix, TSG101, CD9, CD81, and HSP70, were analyzed from EV samples using Western blot analysis. Immunoblotting of ADSC lysates and EV samples for the non-EV component of calnexin was analyzed. **D** The stock solution FBS was diluted with DPBS to a final concentration of 10% FBS. The particle concentration of EVs from 10% FBS was analyzed by NTA. This was the analysis result of the three samples. The difference was considered statistically significant if *p* values were less than 0.05. *P* values less than 0.05 are denoted by by “*”, *p* values less than 0.01 are denoted by “**”, and *p* values higher than 0.05 are denoted by “ns” (non-significant)

Further, EVs were isolated using UF to compare the production rate of the different source cells, including ADSCs and WJ-MSCs. The particle concentration and size were measured at different passages using NTA. The serum-free condition could produce approximately 2000 particles/per ADSC cell and secrete approximately 3500 particles/per WJ-MSC cell (Fig. 4A). The data also suggested that WJ-MSCs could produce more EVs than ADSCs. The average sizes of ADSC-derived EVs were 150 ± 39 nm, whereas the average diameters of WJ-MSC-derived exosomes were 123 ± 14 nm (Fig. 4B). EVs were also examined for the expression of the representative proteins. The results confirmed the presence of MSC-derived EV-enriched markers, such as the biogenesis-related proteins Alix (95 kDa) and TSG101 (45 kDa), HSP70 (70 kDa), CD9 (24 kDa), and CD81 (25 kDa), using Western blot analysis. Moreover, the endoplasmic reticulum protein calnexin was present in cell lysates, but absent in EVs (Fig. 4C). These data suggest most EVs may be exosomes.

In addition, FBS was diluted to 10% with sterile water, and the EV concentration was analyzed using NTA. Approximately 4.4 × 10^8^ to 6.8 × 10^8^ EVs/mL of 10% serum were obtained, indicating the EV concentration in the culture condition. Thus, about 5 × 10^8^ particles/mL were used to test the differentiation capacity of MSCs (Fig. 4D).

### The EVs maintained their chondrogenic differentiation ability

**Fig. 5:**
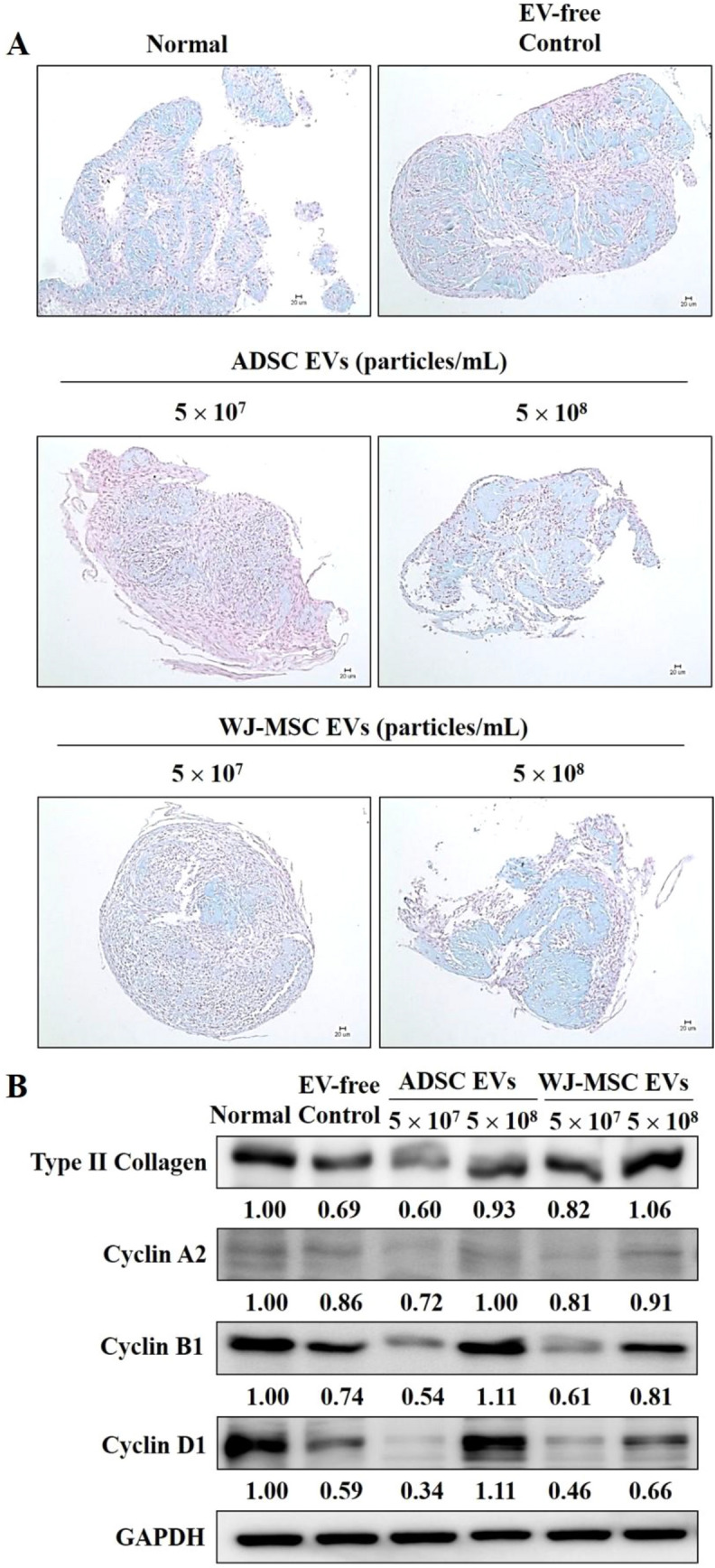
Effects of EVs on stem cell differentiation into chondrocytes. Normal FBS was added to the culture medium used for the MSC differentiation process in the regular condition group (Normal). Cells from the EV-free control group were maintained in medium with 10% EV-depleted FBS during the differentiation process. Cells from the EV test groups were cultured in a medium containing EV-deleted FBS. Cells were added with EVs derived from ADSCs and WJ-MSCs in chondrogenic differentiation medium. The EV concentrations were 5 × 10^7^ and 5 × 10^8^, respectively. The medium was completely replaced every 3 days. **A** Cells were cultured in chondrogenic differentiation medium for 21 days. Tissue sections were stained and observed by microscopy, followed by Alcian blue staining. **B** On day 14, cells were harvested, and lysis buffer was used to extract the proteins. Type II collagen and cyclins (A2, B1, and D1) protein expression levels were assessed by Western blot analysis. Further ImageJ analyses of the bands were performed to measure the protein expression levels

Experiments were conducted to verify whether or not EVs isolated from serum-free culture medium could also promote chondrogenic differentiation capacity. In functional EV experiments, ADSCs were cultured in EV-free FBS medium, and cells were added with different concentrations of EVs derived from ADSCs and WJ-MSCs. At 21 days, the ability of ADSCs to differentiate into chondrocytes was observed. Moreover, treatment in EV-free conditions resulted in a decreased differentiation capacity compared to treatment in FBS-added medium, which served as the normal group*.* However, incubation of ADSCs at 5 × 10^8^ particles/mL EV concentration almost restored GSG and GP production (Fig. 5A).

At 14 days, the expression of chondrogenic differentiation and cell proliferation-related proteins in the early stage was determined. During the chondrogenic differentiation process of ADSCs, the high concentration of MSC-derived EVs (5 × 10^8^ particles/mL) seemed to increase COLII expression compared to 5 × 10^7^ particles/mL. EVs enhanced the chondrogenic differentiation of ADSCs via COLII upregulation (Fig. 5B), indicating that EVs could promote early chondrogenic differentiation *in vitro*. Cellular proliferation requires passage through a cell cycle to produce new cells. The cell proliferation status and the expression of the cell cycle-related proteins cyclins A, B, and D were examined. During differentiation induction, the results showed cyclin A2 and cyclin D1 upregulation after EV treatment, which contributed to the stimulation of DNA synthesis. Treatment of ADSCs with EVs also triggered cyclin B protein expression (Fig. 5B). That is to say, cells retained their ability to divide with the addition of EVs, thereby allowing growth during cell differentiation.

**Fig. 6:**
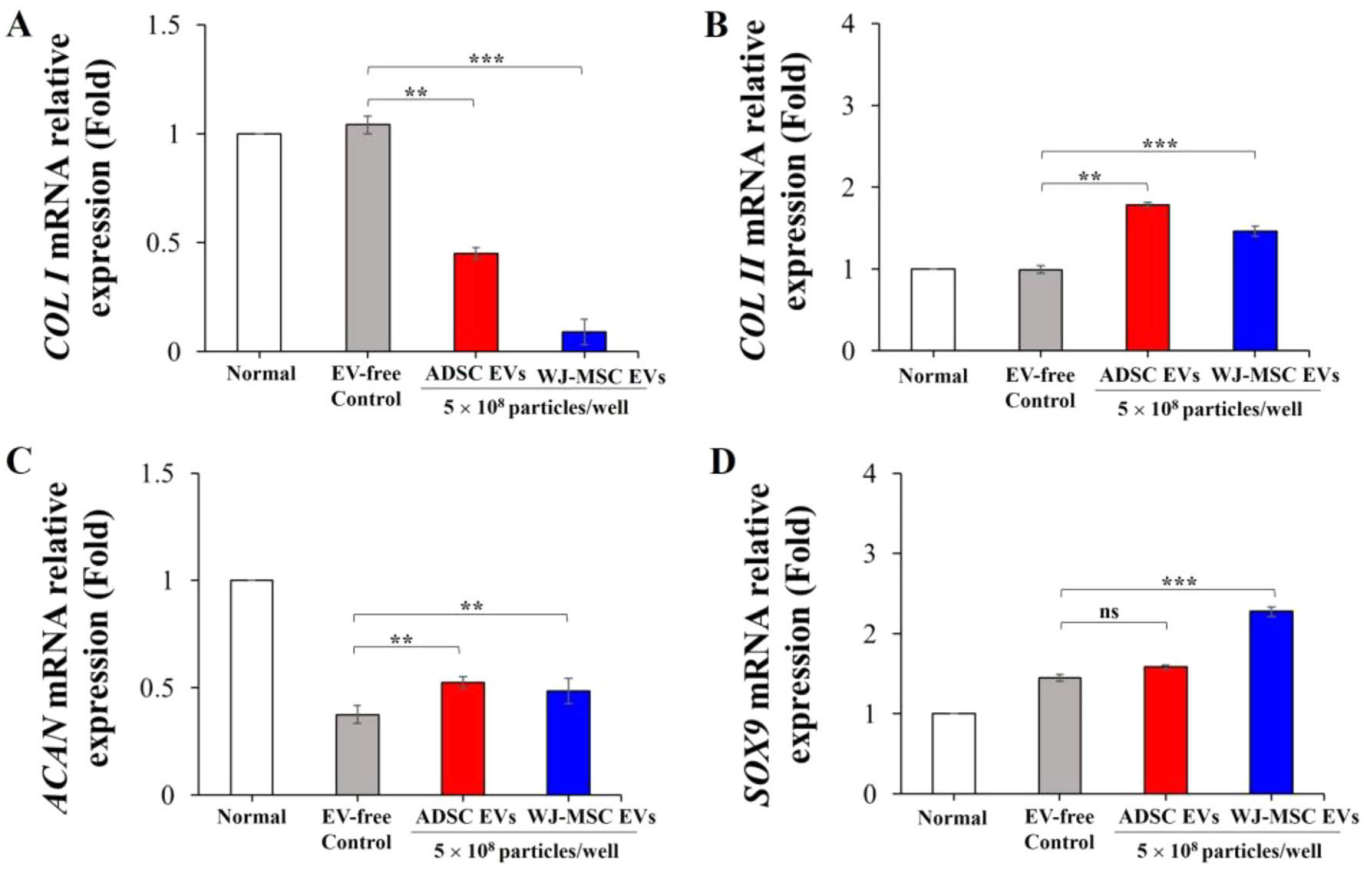
Quantitative real-time gene expression analysis of chondrogenic-specific genes. Normal FBS was added to the culture medium used for the MSC differentiation process in the regular condition group (Normal). Cells from the EV-free control group were maintained in medium with 10% EV-depleted FBS during the differentiation process. Cells from the EV test groups were cultured in a medium containing EV-deleted FBS. Cells were added with EVs derived from ADSCs and WJ-MSCs in chondrogenic differentiation medium. The mRNA expression levels of (**A**) COLI, (**B**) COLII, (**C**) ACAN, and (**D**) SOX9, were measured in chondrogenic differentiated tissues after 21 days. GAPDH was used as an internal control. The value of fold change was normalized to the regular culture condition, and all values are expressed as mean ± SEM, n = 3. *p < 0.05; **p < 0.01, compared to the EV-free group using t-test. A p value higher than 0.05 is denoted by “ns” (non significant)

In addition, we analyzed whether treatment with MSC-derived EV altered mRNA expression of collagen type I (COLI), COLII, aggrecan (ACAN), and SOX9 during chondrogenic differentiation (Fig. 6). The results showed that under treatment with either ADSC-derived EVs or WJ-MSC-derived EVs, both types could reduce COLI mRNA expression levels, while enhancing mRNA expression levels of COLII, ACAN, and SOX9 compared to the EV-free condition. These data suggest that EVs could upregulate the expression levels of chondrogenesis-related genes to promote chondrogenic differentiation.

## MSC-derived EVs reduce lipid droplet formation

**Fig. 7:**
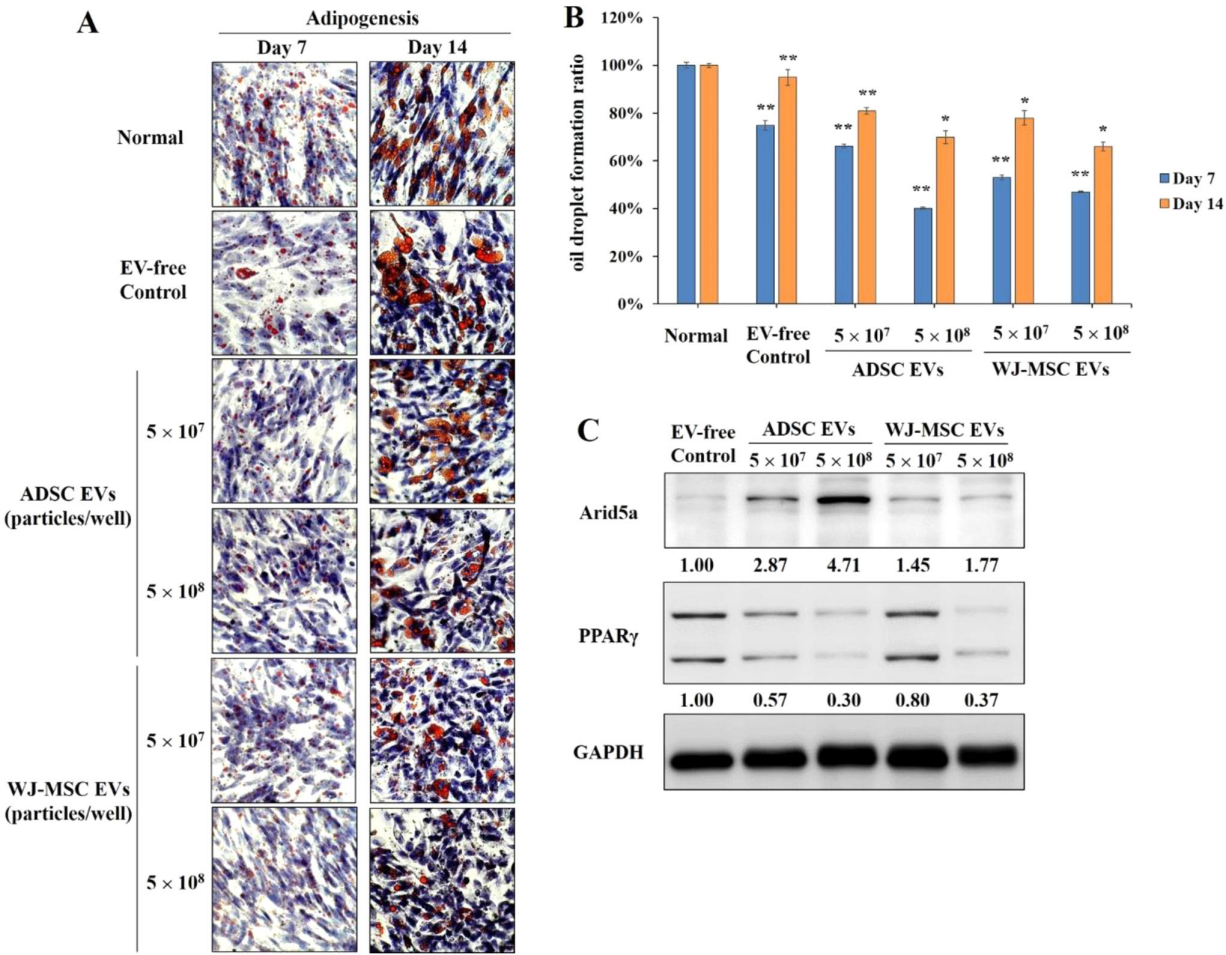
Effects of EVs on stem cell differentiation into adipocytes. During the differentiation process, EVs derived from ADSCs and WJ-MSCs were added to adipogenic differentiation medium and treated with different EV concentrations. The medium was replaced every 3 days. **A** On days 7 and 14, lipid accumulation during adipogenic induction was monitored by Oil Red O staining. Hematoxylin-stained cell nuclei were stained purplish blue. **B** Lipid droplets were dissolved in methanol, and the amount of oil droplet generation was quantified. **C** On day 14, cells were harvested, and lysis buffer was used to extract the proteins. Arid5a and PPARγ protein expression levels were assessed by Western blot analysis. Further ImageJ analyses of the bands were performed to measure the protein expression levels. The ratios of Arid5a and PPARγ protein expression were normalized to GAPDH. *p < 0.05; **p < 0.01, compared to the normal group using *t*-test

Experiments were performed to examine whether or not EVs secreted from the cell culture medium affect fat production. In the differentiation process, the effects of the adipogenic ability of both EVs from FBS and the addition of EVs derived from different MSCs were compared. The staining results showed that MSC-derived EVs could significantly mitigate the volume of the oil droplets (Fig. 7A). The quantitative results also indicated that EV extracted from the stem cell culture medium could alleviate lipid accumulation (Fig. 7B). Thus, these results confirmed that both ADSC- and WJ-MSC-derived EVs decreased the adipogenic induction ability, but both ADSC- and WJ-MSC-derived EVs did not completely inhibit lipid production. We also used ADSCs from another donor as target cells for the chondrogenic and adipogenic differentiation assay to avoid donor effects (Supplementary Fig. S2). The data indicate that both ADSC-derived EV and WJ-MSC-derived EV could not only successfully promote chondrogenic differentiation, but could also reduce lipid production in the second donor's ADSCs.

Furthermore, we explored the expression of adipogenesis-related proteins, including AT-rich interactive domain 5a (Arid5a) and PPARγ. Arid5a is a negative regulator during adipogenic differentiation and it inhibits adipogenesis by inhibiting the transcription of PPARγ [40]. The results showed that treatment with MSC-derived EVs significantly reduced the expression levels of PPARγ, and the protein expression levels of Arid5a were upregulated (Fig. 7C). In summary, both ADSC-derived EVs and WJ-MSC-derived EVs could not only upregulate chondrogenesis-related protein expression levels, but could also downregulate adipogenesis-related protein expression levels, which could result in promotion of MSC differentiation to chondrocytes.

## Discussion

The use of serum-free conditions to collect EVs is widespread in both basic biology and regenerative medicine. In this study, we demonstrated that using EV-depleted FBS culture ADSC reduced differentiation ability. Interestingly, addition of exogenetic MSC-EV, both ADSC and WJ-MSC, not only promoted chondrogenesis, but also reduced adipogenesis *in vitro*. Identification of EVs, which provide rapid chondrogenesis ability when the source cell still maintains a healthy cell phenotype, would be greatly advantageous for quality control of EVs used for osteoarthritis (OA) treatment. Our data suggest that the use of ADSC- and WJ-MSC-derived EVs for chondrocyte regeneration could be beneficial applications in regenerative medicine. We further determined that both ADSC- and WJ-MSC-derived EVs could upregulate expression of chondrogenic-related genes and downregulate expression of adipogenic-related genes to trigger chondrogenesis.

Currently, the top three tissue sources of MSCs used for exosome research are bone marrow (BM), umbilical cord tissue, and adipose tissue [41]. BM-MSCs have higher ALP activity and calcium deposition ability, which could promote cartilage formation and osteogenesis, but BM-MSCs have the disadvantages of senescence within early passages and limited proliferation ability [42–44]. The age of the bone marrow donor also affects the quality of stem cells and influences the chondrogenic response [45]. However, ADSCs are also a candidate cell source regardless of autologous or allogeneic cell therapy in current clinical trials [46]. ADSCs can be generated in large numbers with minimal culture and have high tissue-specific differentiation capabilities. WJ-MSCs, which are obtained from the umbilical cord, are another type of MSC, and they have greater proliferation rates and differentiation potential than other adult MSCs [47]. Therefore, ADSCs and WJ-MSCs were selected as cell sources for preparation of EVs in this study.

In the past few years, the separation of exosomes has been achieved via various techniques, such as ultracentrifugation (UC), UF, size-exclusion chromatography (SEC), and tangential flow filtration system (TFF) [48]. For the EV purification process, this study used a 100 kDa cut-off for UF for the isolation and concentration of EVs from large-scale biological fluids to small volumes. Using NTA, the average particle size of EVs derived from ADSCs or WJ-MSCs was within a diameter of 150 nm. As shown in Fig. 4, the EV generation rate of WJ-MSCs was higher than that of ADSCs. In contrast, WJ-MSC may be a better choice as a tool for mass production of EVs. Because the purity of EVs is also a critical criterion for quality control, the presence of typical exosome markers, such as Alix, HSP70, TSG101, CD9, and CD81, was further verified using Western blot analysis to validate the establishment of a good model for EV production. However, the development of clinical-grade EVs, sterile generation, and high-scale and efficient production are major bottlenecks in the advancement of EV-based therapy.

Comparisons of human ADSCs in different media, i.e., proliferation media and differentiation media, have been conducted. The results showed that even when cells are cultured in a proliferation medium, they also display spontaneous differentiation [49]. Thus, we compared the differentiation potential of chondrogenesis and adipogenesis, but not osteogenesis, in human ADSCs under different conditions in this study.

This study evaluated the efficacy of isolation and production of MSC-derived EVs and further demonstrated the key role of EVs in stem cell differentiation. The results of this study showed that EVs produced from healthy donors markedly enhanced chondrogenesis and attenuated lipid production properties. However, the underlying mechanism by which EVs affect differentiation remains unclear. It has been reported that several microRNAs (miRNAs) and transcription factors could modulate cell proliferation and differentiation. Currently, miRNAs are known to act as activators of adipogenesis, including miR-199, miR-210, miR-378, miR-30b/c, miR-455, and miR-32 [50]. However, some miRNAs block expression of master regulators of adipogenesis, such as miR-27 [51, 52], miR-34a, miR-133, miR-155 [53]. Among them, miR-27 is worth noting. It has been shown that overexpression of miR-27 resulted in specific inhibition of adipogenic differentiation with the blockade of PPARγ and C/EBPα during early onset of adipogenesis [54]. Moreover, miR-27a has been reported to increase osteogenesis [55] and decrease adipogenesis via targeting of myocyte enhancer factor 2C (Mef2c) [56], and miR-27a was downregulated in osteoporosis patients [57]. In addition, animal experiments in rats with knee osteoarthritis showed that upregulation of miR-27a inhibited cartilage collagen destruction and synovial angiogenesis by inhibiting polo-like kinase 2 (PLK2) [58]. In this study, additional EVs did not affect osteogenic differentiation, but promoted chondrogenesis and inhibited adipogenesis. Thus, the additional EVs may be involved in regulating the differentiation of ADSCs through miR-27.

Previous studies have confirmed that EVs are not only capable of enhancing the differentiation of proliferating neural stem progenitor cells, but can also trigger cell differentiation in a dose-dependent manner [59]. In the functional EV assay, the low concentration of MSC-derived EVs did not effectively induce ADSC differentiation. Therefore, during stem cell differentiation, the added EV concentration must be at least equivalent to the EV content under regular growth conditions to effectively trigger the function of cartilage regeneration. If EVs are used as an enhancer in stem cell therapy, the addition of MSC-derived EVs may have an additive effect.

Many studies have previously indicated that excess weight and obesity in young people increase joint loading and contribute to OA progression [60, 61]. Recently, numerous studies have reported that exosomes mitigate metabolic disorders. In animal experiments, MSC-derived exosomes have been reported to alleviate type 2 diabetes, adipose inflammation, and obesity [62, 63]. After intravenous injection of brown adipose tissue-derived exosomes, mice also had reduced body weight, adipocyte sizes, and fatty liver. In this study, the cell test results also showed that EVs derived from MSCs also had the ability to reduce lipid production by stem cells, suggesting that EV therapy may contribute to mitigating metabolic syndrome.

In summary, this study confirmed that EVs play an essential role in stem cell differentiation. An *in vitro* production model of functional exosomes from MSCs was also established. In addition, both ADSC-derived EVs and WJ-MSC-derived EVs triggered MSC differentiation to chondrocytes via upregulating chondrogenic-related gene expression and downregulating gene expression patterns of adipogenesis. With the aid of this production model, MSC-derived EVs may have potential in future clinical applications for cartilage regeneration and could be a promising new therapeutic agent for OA.

## Supplementary Information

Below is the link to the electronic supplementary material.

Supplementary file 1 (PDF 328 kb)
