# Supplementary material for: Mesenchymal Stem Cell Secreted-Extracellular Vesicles are Involved in Chondrocyte Production and Reduce Adipogenesis during Stem Cell Differentiation
Source: Tissue Eng Regen Med. 2022 Nov 8;19(6):1295–310. doi: 10.1007/s13770-022-00490-0 (PMC9679102; doi:10.1007/s13770-022-00490-0)
Supplement: Supplementary file 1 — (PDF 328 kb) [file 13770_2022_490_MOESM1_ESM.pdf]

Supplementary information

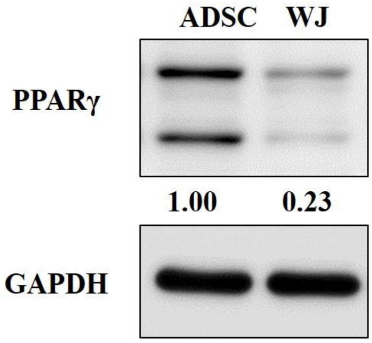

Supplementary Fig. 1

Adipogenic capacity of mesenchymal stem cells from different tissue sources. Two different tissue-derived mesenchymal stem cells, ADSCs and WJ-MSCs, were cultured in adipogenic differentiation medium for 14 days, and then the Western blot method was used to observe the amount of PPARγ protein in ADSCs and WJ-MSCs. Further ImageJ analyses of the bands were performed to measure the protein expression levels. The ratios of PPARγ protein expression was normalized to GAPDH.

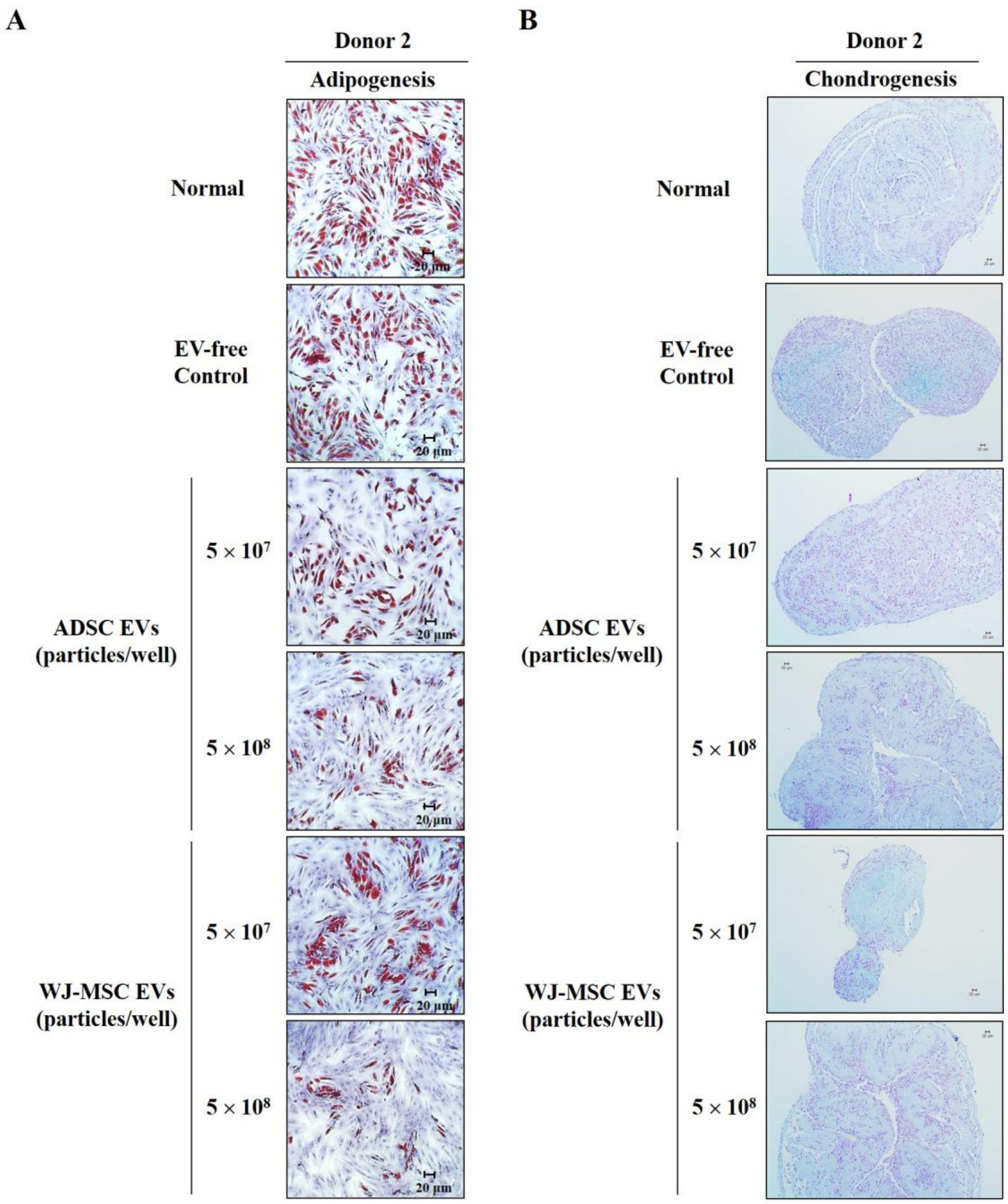

Supplementary Fig. 2

The second donor's adipogenic differentiation and chondrogenic differentiation experiments. **A** On days 14, lipid accumulation during adipogenic induction was monitored by Oil Red O staining. Hematoxylin-stained cell nuclei were stained purplish blue. **B** Cells were cultured in chondrogenic differentiation medium for 21 days. Tissue sections were stained and observed by microscopy, followed by Alcian blue staining.
